# Technological advances in imaging and modelling of leaf structural traits: a review of heat stress in wheat

**DOI:** 10.1093/jxb/eraf070

**Published:** 2025-03-04

**Authors:** Jing He, Kun Ning, Afroz Naznin, Yuanyuan Wang, Chen Chen, Yuanyuan Zuo, Meixue Zhou, Chengdao Li, Rajeev Varshney, Zhong-Hua Chen

**Affiliations:** School of Science, Western Sydney University, Penrith, NSW 2751, Australia; School of Science, Western Sydney University, Penrith, NSW 2751, Australia; School of Science, Western Sydney University, Penrith, NSW 2751, Australia; School of Science, Western Sydney University, Penrith, NSW 2751, Australia; School of Science, Western Sydney University, Penrith, NSW 2751, Australia; Triticeae Research Institute, Sichuan Agricultural University, Chengdu, Sichuan Province 611130, China; School of Science, Western Sydney University, Penrith, NSW 2751, Australia; Triticeae Research Institute, Sichuan Agricultural University, Chengdu, Sichuan Province 611130, China; Tasmanian Institute of Agriculture, University of Tasmania, Launceston, TAS 7250, Australia; Western Crop Genetics Alliance, Centre for Crop and Food Innovation, WA State Agricultural Biotechnology Centre, Food Futures Institute, Murdoch University, Murdoch, WA 6150, Australia; Western Crop Genetics Alliance, Centre for Crop and Food Innovation, WA State Agricultural Biotechnology Centre, Food Futures Institute, Murdoch University, Murdoch, WA 6150, Australia; School of Science, Western Sydney University, Penrith, NSW 2751, Australia; University of Sydney, Australia

**Keywords:** Artificial intelligence, image processing, leaf anatomy, machine learning, microscopy, phenotyping, *Triticum aestivum* L

## Abstract

Abiotic stresses such as heat waves significantly reduce wheat productivity by altering leaf anatomy and physiology, leading to reduced photosynthetic carbon assimilation and crop yield. Despite the advancement in various imaging technologies at the field, canopy, plant, tissue, cellular, and subcellular levels, phenotyping of imaging-based leaf structural traits (e.g. vein density, stomatal density, and stomatal aperture) for abiotic stresses is still time-consuming and expensive without the aid of artificial intelligence (AI) and machine learning (ML). This review consolidates current knowledge of wheat leaf structural and functional adaptations to heat stress and highlights key advancements in imaging technologies for studying these important phenotypic traits. Recent high-resolution, non-destructive imaging technologies, including confocal laser scanning microscopy, X-ray computed tomography, and optical coherence tomography, have enabled *in vivo* visualization of plants. Integrating these imaging techniques with AI/ML facilitates high-throughput phenotyping and the modelling of stress responses. We emphasize the potential for future research to leverage these technological advancements in imaging and AI, combining imaging data with physiological and multi-omics studies to deepen the understanding of plant heat tolerance mechanisms. Such multidisciplinary integration in leaf structure phenotyping will accelerate the development of resilient wheat varieties, offering critical insights for crop improvement in the face of climate change.

## Teaser

This review provides an integrated analysis of wheat leaf adaptations to heat stress, showcasing cutting-edge image phenotyping technologies and their potential to inform future research on crop resilience.

## Introduction

Wheat (*Triticum aestivum* L.) is one of the most important staple crops worldwide, contributing approximately 20% of the total calories and protein consumed by humans (Shiferaw *et al.*, 2013). Although cultivated in diverse agro-ecological environments, wheat productivity is highly sensitive to abiotic stresses, particularly elevated temperatures. Agricultural models predict up to a 6.4% decline in global wheat yield for every 1 °C increase in temperature (Liu *et al.*, 2016). Moreover, the frequency, intensity, and duration of heatwaves are expected to rise in key wheat-producing regions, including China, the USA, France, and Australia, threatening yield stability and global food supplies (Brown, 2020; Perkins-Kirkpatrick and Lewis, 2020). Developing heat-resilient wheat varieties is therefore critical and requires a deeper understanding of how heat stress impacts the plant at multiple levels, from cellular physiology to tissue and organ structure.

Heat stress can disrupt wheat development, particularly during sensitive stages such as flowering and grain filling (Farooq *et al.*, 2011). Temperatures exceeding 30 °C during these stages can induce pollen abortion (Shenoda *et al.*, 2021) and shorten grain-filling duration (Ullah *et al.*, 2022), resulting in reduced yield, grain weight, and quality (Chileshe *et al.*, 2023). These physiological disruptions often manifest through structural changes in leaves—the primary organs for photosynthesis and transpiration regulation—highlighting the importance of cell- and tissue-level adaptations to heat stress in plants (Lal *et al.*, 2022; Rascio *et al.*, 2023).

The anatomy of wheat leaves plays a central role in modulating the plant’s response to heat stress (Ouyang *et al.*, 2017; Zahra *et al.*, 2023). The epidermal layer, which contains stomata (the microscopic pores that regulate water loss and gas exchange), often undergoes significant changes under elevated temperatures (Yuan *et al.*, 2020). Heat stress can alter stomatal density, aperture, and distribution patterns to minimize dehydration while optimizing water use efficiency (Feng *et al.*, 2014). In addition to stomatal adjustments, other anatomical changes at the tissue level include increased cuticular wax accumulation, mesophyll cell restructuring, and modifications to vascular bundles, all of which contribute to improved water retention and cooling capacity (Rascio *et al.*, 2023).

In uncovering these leaf structural changes, microscopy and imaging technologies play crucial roles. Traditional microscopy techniques, such as light microscopy (LM), scanning electron microscopy (SEM), transmission electron microscopy (TEM), and fluorescence microscopy, have provided critical insights into leaf anatomy under stress (Lersten, 1987). However, these techniques often rely on destructive sampling and are limited to static observations (Harwood *et al.*, 2020; Yuan *et al.*, 2020), restricting dynamic studies over the time course of heat stress. The recent technological advancements of confocal laser scanning microscopy (CLSM) (Rath *et al.*, 2014), X-ray computed tomography (CT) (Mathers *et al.*, 2018), and optical coherence tomography (OCT) (de Wit *et al.*, 2020) enable more detailed, non-invasive visualization of leaf tissues under heat stress. The live tracking and monitoring of stomatal dynamics with portable microscopes or phenotyping platforms further complements these tools, offering opportunities to simultaneously monitor physiological traits such as water status and stomatal regulation (Toda *et al.*, 2021; Sai *et al.*, 2023). These imaging techniques, particularly when used in controlled heat stress experiments, provide critical insights into the dynamic anatomical changes that underpin plant resilience.

The integration of advanced imaging tools with artificial intelligence (AI) and machine learning (ML) technologies can further transform how plant anatomical data are analysed. AI refers to systems that mimic human intelligence to perform complex tasks, such as data analysis and prediction, while ML is a subset of AI that allows machines to learn patterns from data and improve their performance over time without explicit programming (Jakhar and Kaur, 2020). Automated phenotyping platforms driven by AI/ML algorithms allow researchers to extract key anatomical traits, such as stomatal density and cell wall thickness, and link them with heat tolerance (Khoshravesh *et al.*, 2022). Predictive models generated from these algorithms also facilitate high-throughput phenotyping, helping to identify promising heat-resilient varieties faster than traditional breeding methods (C. Zhu *et al.*, 2021; Pathoumthong *et al.*, 2023). However, challenges remain, including the need for high-quality imaging data, standardized analytical pipelines, and the biological validation of AI-based predictions.

This review aims to provide an overview of the current understanding of wheat leaf anatomical responses to heat stress, with a focus on cell-, tissue-, and organ-level changes. We then explore both current and advanced imaging methodologies, highlighting how these tools have advanced the study of leaf anatomy. Additionally, we discuss the potential of integrating imaging technologies with AI/ML to develop predictive models for assessing plant performance under heat stress. By synthesizing recent findings and identifying research gaps, this review also offers insights into future directions for leaf structure studies and their role in crop improvement programmes targeting heat resilience.

## Leaf structural and functional adaptations to heat stress in wheat

Heat stress has profound effects on the structural and functional integrity of wheat leaves, which serve as primary organs for photosynthesis, transpiration, and gas exchange. This section explores the intricate anatomy of wheat leaves and their adaptive responses to elevated temperatures.

### Leaf anatomy and heat stress response in wheat

The anatomy of leaves is intricately structured to optimize photosynthesis, water transport, and gas exchange, processes essential for plant growth and yield. Leaves are composed of three primary tissue layers: the epidermis, mesophyll, and vascular bundles. The epidermis serves as the outermost protective layer, featuring a waxy cuticle that minimizes water loss and provides a physical barrier against pathogens and UV radiation (Arya *et al.*, 2021). The cuticle is enriched with epicuticular waxes, which vary in chemical composition among wheat varieties, playing a pivotal role in water retention and heat tolerance (Bi *et al.*, 2017). In addition to the cuticle, the epidermis contains specialized structures like stomata and trichomes. Stomata are composed of two guard cells and flanking subsidiary cells, and they facilitate gas exchange and transpiration. They are distributed in rows on both leaf surfaces, with greater density on the adaxial side (Toda *et al.*, 2021). The regulation of stomatal aperture is crucial for balancing CO_2_ uptake and water conservation, especially under heat stress (Pathoumthong *et al.*, 2023). Trichomes, which are non-glandular and simple in wheat, aid in reducing transpiration and reflecting excess solar radiation, contributing to thermoregulation (Hakeem *et al.*, 2021).

Beneath the epidermis lies the mesophyll cells, rich in chloroplasts for photosynthesis, and the intercellular air spaces facilitating CO_2_ diffusion (Ouyang *et al.*, 2017). Mesophyll cell size and chloroplast number vary between wheat species and varieties, with modern hexaploid wheat possessing larger cells and more chloroplasts. This evolution enhances their photosynthetic potential compared with the ancestral species (Wilson *et al.*, 2021). The vascular bundles, consisting of xylem and phloem, are embedded within the mesophyll and play critical roles in water and nutrient transport. They are surrounded by bundle sheath cells and are densely arranged to support efficient hydraulic conductivity and mechanical stability (Leegood, 2008; Harwood *et al.*, 2020). In wheat, the main vascular bundles are typically concentrated along the midrib, with smaller veins distributed evenly throughout the leaf blade to optimize nutrient distribution and water flow (Hu *et al.*, 2005). The high density of vascular bundles in wheat leaves correlates with increased hydraulic conductance and photosynthetic efficiency (Brodribb *et al.*, 2007), providing a crucial link to agronomic performance.

### Morphological and physiological changes in wheat leaves under heat stress

**Fig. 1.**
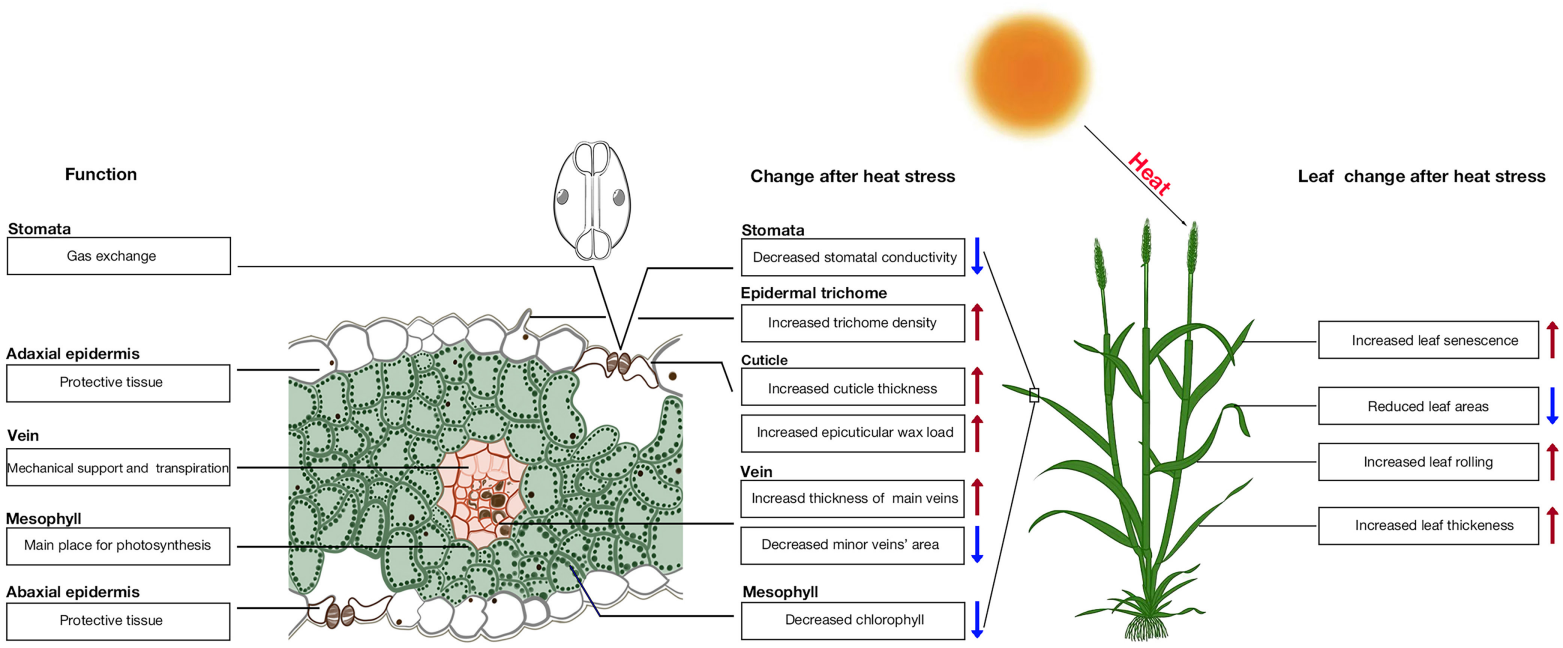
Morphological and anatomical changes in wheat leaf structure under heat stress. The arrow colours indicate the direction of trait changes following heat stress: red arrows signify an increase, while blue arrows indicate a decrease.

While the anatomical features of wheat leaves are finely tuned for photosynthesis and water regulation, elevated temperatures induce significant disruptions to these structures, affecting their functional efficiency (Fig. 1). For example, heat stress significantly impacts wheat stomatal traits by typically triggering stomatal closure to conserve water, but this comes at the compromise of reduced photosynthesis and CO_2_ assimilation (Sun *et al.*, 2021). Some heat-tolerant wheat varieties could maintain higher stomatal conductance under elevated temperature, which sustains transpiration and cooling, thus mitigating heat damage (Hakeem *et al.*, 2024). Changes in stomatal density were also observed under heat stress; higher density may enhance transpiration rates to cool down the leaves (C. Zhu *et al.*, 2021). The production and composition of epicuticular waxes on the leaf surface can also be altered under heat. An increase in wax deposition enhances reflectance and reduces water loss, thereby maintaining lower leaf temperatures (Mohammed *et al.*, 2018; Willick *et al.*, 2018). It was reported that heat-stressed wheat accumulates specific long-chain hydrocarbons and fatty alcohols, contributing to better heat tolerance (Mondal *et al.*, 2015; Wang *et al.*, 2015). Similarly, increase of trichome density can further aid in thermoregulation and minimizing water loss (Hakeem *et al.*, 2021; Saska *et al.*, 2021). These structural adaptations are essential for regulating water flow and nutrient distribution under stress conditions (Rascio *et al.*, 2023).

However, the morphological and physiological disruptions caused by heat stress often compromise overall plant performance (Fig. 1). At the organ level, one of the most apparent morphological changes is a reduction in leaf area. Smaller leaves have thinner boundary layers, promoting heat dissipation and reducing the risk of overheating (Zanella *et al.*, 2023). Chen *et al.* (2018) found heat stress accelerated leaf senescence and decreased leaf area index of winter wheat. This adaptation, while beneficial for thermal regulation, comes at a cost of reduced surface area, which can lower overall carbon assimilation and eventually grain yield (Kumar *et al.*, 2022). In addition to reductions in leaf area, elevated temperature also impacts internal leaf structures such as thickness and cellular composition. It was reported that heat-stressed wheat leaves often develop thicker mesophyll layers, which may be due to cell wall modifications and increased cell turgor as a defence mechanism (Ouyang *et al.*, 2017). A positive correlation between leaf thickness, relative chlorophyll, and grain yield were also reported by Chileshe *et al.* (2023); this correlation was found to be stronger in heat-tolerant wheat genotypes. Leaf angle, defined as the inclination between the leaf midrib and the stem, is a key factor in modulating light interception and heat dissipation. Heat stress often induces a reduction in flag leaf angle, resulting in a more erect posture that reduces excessive solar radiation and minimizes overheating (Mantilla-Perez and Salas Fernandez, 2017; Li *et al.*, 2023). This adaptation is particularly beneficial for breeding programmes aimed at optimizing canopy architecture to balance light use efficiency and thermal tolerance in wheat varieties.

Heat stress also induces profound changes at the cellular and subcellular level. Cell walls often exhibit increased rigidity due to heat-induced lignin accumulation; this change enhances structural stability but may hinder cell expansion and flexibility (Bala *et al.*, 2016; Ouyang *et al.*, 2017). In mitochondria, elevated temperature disrupts the electron transport chain, leading to the overproduction of reactive oxygen species (ROS), which damages mitochondrial membranes and affects ATP synthesis (Scafaro *et al.*, 2021; Lal *et al.*, 2022). Prolonged exposure to heat often disrupts organization, ultrastructure, and biochemistry of chloroplasts, leading to impaired light absorption and reduced electron transport efficiency and photosynthetic rate (Feng *et al.*, 2014; Zahra *et al.*, 2023; Aliprandi *et al.*, 2024). Additionally, enzymes crucial for carbon fixation, such as Rubisco, are heat-sensitive and undergo denaturation at high temperatures, exacerbating the decline in photosynthetic efficiency (Degen *et al.*, 2021; Carmo-Silva and Sharwood, 2023). As a result, heat stress induces leaf senescence, a process marked by chloroplast degradation, vacuole membrane collapse, and loss of membrane integrity (Lal *et al.*, 2022). The breakdown of chlorophyll and the inhibition of enzyme activities collectively disrupt carbon assimilation and plant growth, consequently reducing grain filling and overall yield potential (Wijewardene *et al.*, 2021; Chileshe *et al.*, 2023; Zanella *et al.*, 2023). The structural and biochemical changes observed at various levels collectively impair photosynthetic efficiency and water use dynamics, leading to reduced biomass and grain yield (Kaur *et al.*, 2021; Shenoda *et al.*, 2021). Genotypic variability offers opportunities for selecting cultivars with adaptive anatomical traits, such as higher leaf area index and stomatal density, or enhanced antioxidant defence mechanisms (Farooq *et al.*, 2011; Ullah *et al.*, 2022).

In summary, wheat leaves undergo significant structural and functional changes under heat stress, affecting photosynthesis, water regulation, and yield. Adaptive traits, such as optimized stomatal conductance and enhanced wax deposition, highlight the potential for breeding heat-tolerant varieties. Therefore, advanced microscopy technologies have become indispensable tools to understand the structural and functional adaptations of wheat leaves under heat stress. Table 1 summarizes these techniques, highlighting their sample preparation requirements, advantages, limitations, and potential applications in this context. These techniques can be used to study the effects of heat stress on the leaves of wheat and other plants, providing transferable insights.

**Table 1.** Traditional and advanced microscopy techniques for wheat leaf analysis

Technique	Sample preparation	Advantages	Limitations	Applications to wheat leaf heat stress study	Reference
Light microscopy	Simple; thin sectioning; staining optional	Easy to use, low cost, suitable for routine analysis	Limited resolution; cannot visualize subcellular structures; potential tissue distortion	General anatomical studies such as leaf thickness, mesophyll cell arrangement, stomatal density, and cuticular features	Jäger *et al.* (2014), Ouyang *et al.* (2017), Dunn *et al.* (2019)
Fluorescence microscopy	Moderate; requires fluorescent dyes or markers for non-fluorescent compounds	High sensitivity and specificity; allows dynamic molecular tracking	Expensive; limited to fluorescently labelled samples or autofluorescent molecules; does not provide ultrastructural detail	Real-time monitoring of reactive oxygen species, calcium ions, and membrane integrity under heat stress; subcellular localization of proteins; chlorophyll fluorescence during early stress response	Fan and Xing (2004), Fedyaeva *et al.* (2014), Feng *et al.* (2014)
Scanning electron microscopy	Requires dehydration and conductive coating	High resolution for surface structures, robust detail	Destructive sample preparation, only surface imaging	Detailed surface structure analysis, such as stomatal patterning, epicuticular wax morphology, and trichome distribution under heat stress	Jäger *et al.* (2014), Willick *et al.* (2018), Yuan *et al.* (2020)
Transmission electron microscopy	Extensive; includes fixation, dehydration, and ultrathin sectioning	Extremely high resolution, reveals fine subcellular details	Time-consuming, requires expert skills	Observes ultrastructural changes in organelles, such as chloroplast ultrastructure, thylakoid membranes, and mitochondrial damage under heat stress	Marutani *et al.* (2014), Horwath *et al.* (2020), Aliprandi *et al.* (2024)
Confocal laser scanning microscopy	Complex; requires fluorescent markers and careful sample preparation for non-fluorescent compounds	Enables live imaging and 3D visualization	Limited imaging depth, high cost; fluorescent markers needed on non-fluorescent molecules	Non-invasive 3D visualization of live tissues, chloroplast arrangement, mesophyll cell structure, and stomatal behaviour under heat stress	Ayadi *et al.* (2011), Lundgren *et al.* (2019), Han *et al.* (2023)
X-ray computed tomography	Minimal	Non-destructive, provides 3D visualization; suitable for whole-plant imaging	Lower resolution for cellular features; potential for tissue damage with prolonged exposure	Non-destructive 3D assessment of vascular architecture, seed morphology, and xylem vulnerability under heat stress	Mathers *et al.* (2018), Schmidt *et al.* (2020), Withers *et al.* (2021)
Optical coherence tomography	Minimal	Non-destructive real-time imaging, high temporal resolution sensitive to structural changes	Limited depth, primarily surface-level imaging	Quantification of cuticle thickness; real-time structural monitoring, such as water transport and tissue deformation	Anna *et al.* (2019), Rajagopalan *et al.* (2020), Licaj *et al.* (2024)
Portable microscopes	Minimal; typically requires intact, live tissues	Real-time monitoring; captures dynamic physiological processes; suitable for functional analysis	Limited to small sample areas; challenging to replicate field conditions; requires precise environmental control	Tracking rapid stomatal closure and reopening, assessing stomatal sluggishness, and evaluating water conservation strategies under heat stress	Karve *et al.* (2015), Sun *et al.* (2021), Toda *et al.* (2021)

## Current approaches of studying leaf anatomy under stress

**Fig. 2.**
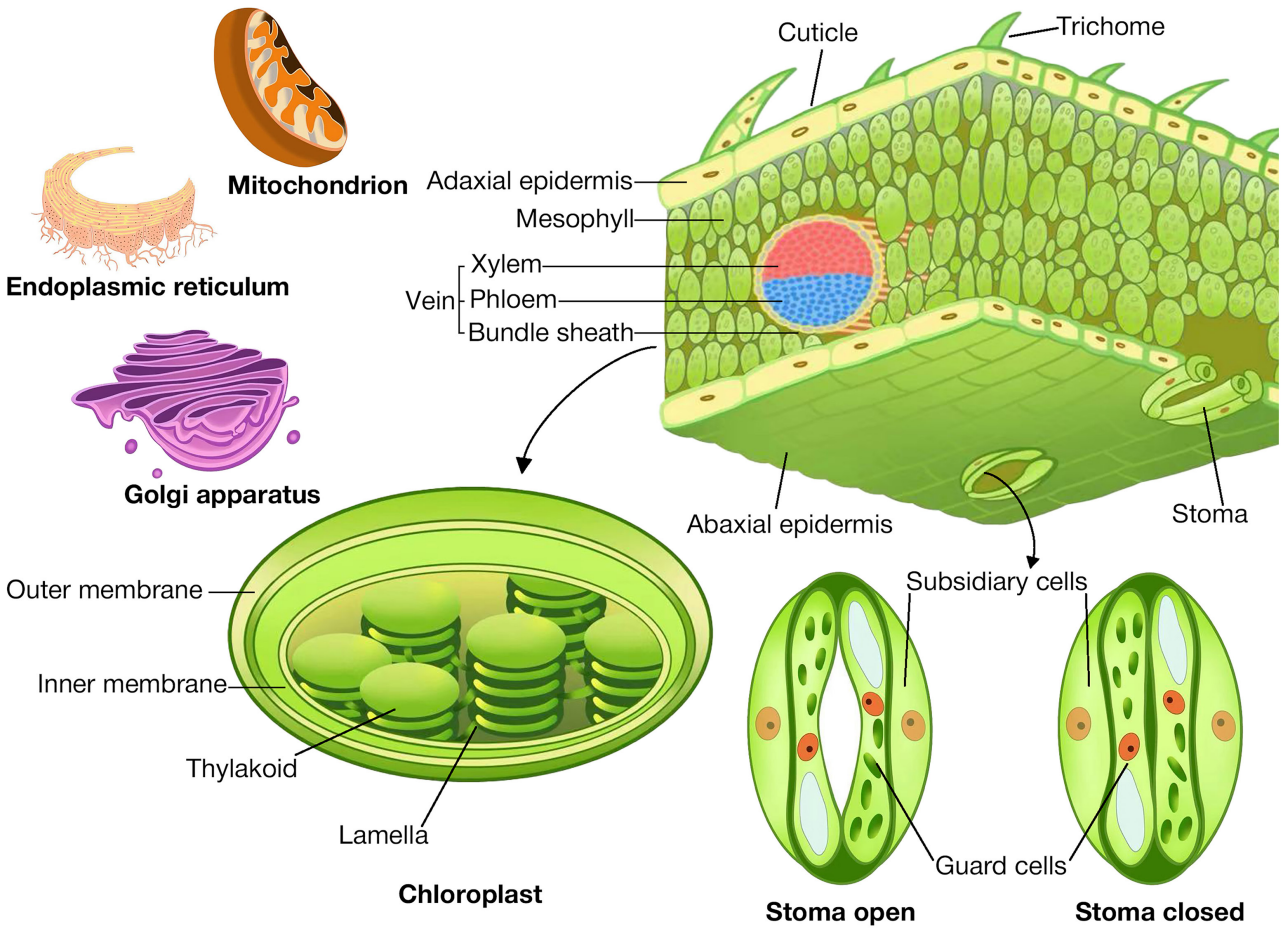
Schematic diagram of the anatomical, cellular, and organelle structures of wheat leaf tissue.

Microscopy and imaging techniques have laid the foundation for understanding the anatomical adaptations of leaves under stress. These methods have provided essential insights into surface morphology, tissue organization, cellular structures, and organelle changes. A detailed depiction of wheat leaf tissues, cellular, and organelle structures that are normally imaged by the current microscopy techniques is provided in Fig. 2. This section explores the principles, applications, recent advancements, and limitations of traditional imaging approaches.

### Light microscopy for general anatomical studies

LM, as the base model of optical microscopy, is a longstanding tool for studying the overall structure of plant tissues. Bright-field LM uses visible light to magnify sectioned tissues and is particularly effective for assessing leaf thickness, mesophyll organization, and vascular structures critical for plant stress responses (Harter *et al.*, 2012; Parrotta *et al.*, 2020; Yan *et al.*, 2021). It has been widely used to study features such as stomata (Dunn *et al.*, 2019; C. Zhu *et al.*, 2021) and epidermal cell size (Hendriks *et al.*, 2022), which influence water regulation under stress. Recent advancements, such as digital imaging and automated software for quantitative trait analysis (Li *et al.*, 2022), have expanded the capabilities of LM for high-throughput studies in leaf anatomy. For instance, Buckner *et al.* (2019) developed an automated image analysis pipeline to track cell division dynamics in Arabidopsis roots. This approach allowed high-resolution, spatiotemporal analysis of cell cycle progression under combined heat and iron deficiency stress, uncovering antagonistic interactions between stress response pathways. In some studies in wheat under heat stress, LM has been used to assess cuticle thickening and bundle sheath cell development—anatomical features that contribute to heat tolerance by improving water conservation and maintaining photosynthetic efficiency (Mohammed *et al.*, 2018).

However, the resolution of LM, limited to 20 nm, restricts its ability to observe subcellular structures such as chloroplast ultrastructures and thylakoid membranes (Jonkman *et al.*, 2020). Also, the sample preparation process can result in tissue distortions and/or artifacts, affecting data accuracy (Diel *et al.*, 2020). While LM can be used to assess basic cuticular features such as thickness, more detailed studies of cuticular morphology, including surface topology, often require advanced techniques such as SEM, TEM, or CLSM (Su *et al.*, 2020; Saska *et al.*, 2021). Albeit this, LM remains indispensable for tissue- and cell-level observations, particularly when coupled with histochemical stains to enhance visualization of cellular components, such as chloroplasts (Zhang *et al.*, 2010) and trichomes (Naidoo *et al.*, 2021; Sarfraz *et al.*, 2024).

To address these limitations, advanced LM methods have been developed, offering higher contrast and improved visualization of unstained tissues. Such improvements, including darkfield illumination, phase-contrast microscopy, polarization microscopy, and differential interference contrast microscopy, have been particularly valuable for live-cell imaging and studies involving subtle structural differences (Khoshravesh *et al.*, 2022). For example, Zhang *et al.* (2020) investigated the penetration and movement of temperature-responsive star polymers in tomato leaves using hyperspectral-enhanced darkfield microscopy. This method provided detailed visualization of polymer interactions with the cuticle and epidermal cell layers, illustrating how darkfield illumination can offer high-contrast imaging of leaf surface features and facilitate the study of agrochemical delivery under different environmental conditions. In another study, Parrotta *et al.* (2020) employed phase-contrast microscopy to examine the cytological impacts of chronic heat stress on tomato leaf cells. This technique revealed subtle changes in cellular structures, such as chloroplast disorganization and lipid body formation, demonstrating its utility for studying heat-induced disruptions in cellular organization in wheat and providing insights into how such damage may influence photosynthetic efficiency. In addition to visualizing cellular structures, techniques such as atomic force microscopy (AFM) provide quantitative insights into the mechanical properties of wheat leaves under heat stress. AFM can measure cell wall stiffness and elasticity at the nanoscale, which are critical for understanding how heat-induced changes impact tissue integrity, leaf expansion, and water retention (Farokh Payam and Passian, 2023; Y. Zhang *et al.*, 2023). For example, wheat leaves exposed to high temperatures may exhibit increased cell wall rigidity due to lignin deposition (Bala *et al.*, 2016), which could be quantified using AFM. Combining such measurements with light microscopy enables a more comprehensive analysis of heat stress adaptations in wheat.

### Scanning and transmission electron microscopy for surface and ultrastructural analysis

SEM and TEM provide unparalleled details of the fine-scale and ultrastructural features of plant tissues. SEM excels in capturing surface characteristics, making it ideal for studying leaf epidermal structures. By directing an electron beam across the sample surface, SEM generates high-resolution images that reveal intricate features such as stomatal patterning (Verma *et al.*, 2020; Nasirzadeh *et al.*, 2022), epicuticular wax structure (Willick *et al.*, 2018), and the topography of epidermal cells and trichomes (Yuan *et al.*, 2020). SEM can achieve magnifications up to 1 000 000 times with a resolution as fine as 1 nm (Rawson *et al.*, 2020). Two recent studies utilizing SEM have demonstrated that heat and drought stress can alter the surface characteristics of wheat leaves, including elevated accumulation of cuticular wax, which enhances leaf reflectance and limits transpiration (Bi *et al.*, 2017; Su *et al.*, 2020). SEM imaging has also revealed that heat- or drought-stressed wheat leaves often exhibit reduced stomatal apertures, a protective response that limits water loss at the expense of photosynthesis (Hakeem *et al.*, 2024; Licaj *et al.*, 2024). However, the SEM process requires samples to be dehydrated and coated with a conductive material, which can alter the natural morphology of leaf surfaces and is time-consuming in sample preparation and measurements (Yuan *et al.*, 2020). Additionally, SEM is primarily a two-dimensional (2D) imaging technique and may not fully capture the three-dimensional (3D) architecture of leaf tissues (Czymmek *et al.*, 2020; Harwood *et al.*, 2020).

Compared with SEM and LM, TEM utilizes the shorter wavelengths of electron beams (100 000 times shorter than those of photons in visible light) to provide significantly higher resolution, capable of revealing sub-nanometre details of cellular structures (Horwath *et al.*, 2020). This makes TEM invaluable for examining ultrastructural features such as the membranes in chloroplasts (Li *et al.*, 2020), mitochondria (Vassileva *et al.*, 2009), and thylakoid (Aliprandi *et al.*, 2024). In wheat, TEM studies have documented thylakoid swelling, disrupted grana stacks, and membrane disintegration under heat stress, indicating severe damage to the photosynthetic machinery (Luo *et al.*, 2010; Marutani *et al.*, 2014; Aliprandi *et al.*, 2024). These findings explain the photosystem II damage and decline in photosynthetic efficiency observed in heat-stressed wheat plants. Albeit its advanced features, TEM has similar issues as SEM; for example, it requires extensive sample preparation, including fixation, dehydration, and sectioning, which makes it time-consuming and technically challenging (Chua *et al.*, 2022). TEM is also limited to the observation of thin sections of tissue, providing a 2D view of complex 3D structures (Zhang *et al.*, 2010; Shen *et al.*, 2024).

Recent advancements in electron microscopy (EM), such as cryogenic EM—involving flash-freezing of samples to temperatures below −150 °C—have improved the ability to observe biological samples in their native state with near-atomic resolutions, minimizing artifacts associated with traditional sample preparation methods (Yu *et al.*, 2021; Chua *et al.*, 2022). It should be noted that the conventional EM images can appear 3D by imaging the sample from different angles, but they do not contain depth information, misleading the estimation of sample volumes (Harwood *et al.*, 2020). New techniques such as serial block-face (SBF)-SEM and focused ion beam (FIB)-SEM have been introduced recently. For instance, SBF-SEM was employed to create 3D reconstructions of leaf cell structures of wheat and chickpea, revealing that traditional 2D cross-sections significantly underestimated chloroplast volume by 45–61% (Harwood *et al.*, 2020). This advancement provides a more accurate understanding of mesophyll and chloroplast architecture, crucial for analysing the effects of heat stress on leaf cellular changes. Moreover, a combined cryo-FIB-SEM was used for visualizing *Chlorella pyrenoidosa* cells in 3D at near-native states (Guo *et al.*, 2022). The images revealed intricate subcellular changes, complementing the proteomic and metabolomic study of carbon fixation in photosynthesis. Thus, these integrated microscopy techniques in conjunction with other multi-omics studies (Zanella *et al.*, 2023) enable a comprehensive analysis of heat response in wheat leaves. They can provide detailed insights into changes in chloroplast ultrastructure, cuticle development, and stress-induced metabolic reprogramming, paving the way for targeted improvements in heat tolerance.

In addition to SEM and TEM, techniques such as Fourier-transform infrared (FTIR) spectroscopy, Raman spectroscopy, and nuclear magnetic resonance (NMR) spectroscopy offer valuable insights into the chemical composition of wheat leaves under heat stress. For example. FTIR spectroscopy can detect chemical changes in the epicuticular wax composition (Willick *et al.*, 2018; Osman *et al.*, 2022). Similarly, Raman spectroscopy can provide spatially resolved data on carotenoid content, enabling the mapping of antioxidant responses (Altangerel *et al.*, 2021), while NMR spectroscopy could help to identify stress-induced modifications in lipids and other metabolites (Coutinho *et al.*, 2024). These complementary methods provide a molecular-level understanding of wheat’s biochemical adaptations to heat stress, which can be integrated with ultrastructural data from electron microscopy.

### Histochemical staining for tissue-specific analysis

To improve the visualization and highlight the chemical composition and structural modifications of plant tissues under stress, histochemical staining is often adopted before microscopic observations (An *et al.*, 2021; Jiang *et al.*, 2021; Yadav *et al.*, 2021). This method involves the application of various stains that selectively bind to specific molecules, such as lignin, cellulose, and phenolic compounds. For instance, toluidine blue and safranin are employed to highlight tissue boundaries and differentiate between cell types (Chen *et al.*, 2021; Yadav *et al.*, 2021). Histochemical staining can provide valuable information about tissue-level changes, such as cell wall composition (Shao *et al.*, 2021) and the accumulation of stress-related metabolites in wheat (Dissanayake *et al.*, 2024). For instance, increased lignin deposition has been observed in heat- and drought-stressed wheat leaves, indicating a potential adaptive response to enhance structural integrity and protection against environmental stresses (Bala *et al.*, 2016). Additionally, histochemical techniques are also widely used to assess the distribution in leaves and roots of phenolic compounds, which are known to play a role in plant defence mechanisms (Rahman and Punja, 2005; Mercado *et al.*, 2025). Staining for ROS in leaf tissues has shown that heat stress induces oxidative damage. This damage is associated with alterations in cellular structure and function. These findings provide valuable insights into the physiological impacts of heat stress on wheat productivity and offer potential targets for breeding heat-resilient wheat varieties (Zang *et al.*, 2017; Lal *et al.*, 2022).

While histochemical staining is primarily applied in light microscopy (Dissanayake *et al.*, 2024), certain stains are also compatible with SEM and TEM, improving contrast for these techniques (Płachno *et al.*, 2024). For example, osmium tetroxide enhances lipid visualization in SEM (Patil *et al.*, 2024), while uranyl acetate (Płachno *et al.*, 2024) and lead citrate (Xu *et al.*, 2021) are used in TEM to highlight membranes and cell walls. Despite its advantages, histochemical staining is inherently destructive and require fixation and sectioning of samples (Yadav *et al.*, 2021). Optimizing staining protocols for specific tissues and stress conditions can be time-consuming. Additionally, stains are limited in their ability to provide dynamic biological information on tissue function, which requires complementary methods for real-time monitoring (Jiang *et al.*, 2021). To overcome these challenges, fluorescent-tagged stains with chemical probes and genetic modifications have been developed, which uses fluorescence and confocal microscopes to increase the specificity of histochemical analysis (Lu *et al.*, 2018).

### Fluorescence-based optical microscopy for early stress detection

Fluorescence-based optical microscopy builds upon traditional staining methods by using fluorophores—dyes or probes that emit light upon excitation under specific wavelengths (Mijovilovich *et al.*, 2020; An *et al.*, 2021). This technique offers higher sensitivity and specificity, enabling the visualization of dynamic cellular processes and tracking of specific molecules and ions, such as ROS (Rossi *et al.*, 2017) and calcium ions (Grenzi *et al.*, 2021), and membrane integrity markers (Wang *et al.*, 2020). Fluorescence microscopy is advantageous for non-invasive monitoring of living tissues, making it suitable for studying real time early stress responses (Van Eeckhout *et al.*, 2021). For example, fluorescence microscopy has been employed to visualize the autofluorescence of phenolic compounds and chloroplasts, as well as to assess cell wall permeability using 4′,6-diamidino-2-phenylindole staining, providing insights into pathogen-induced cellular responses (Szechyńska-Hebda *et al.*, 2013).

In wheat, fluorescence microscopy has been used to study the subcellular localization of heat-responsive proteins, adding valuable insights to molecular and physiological analyses (Han *et al.*, 2024). However, fluorescence microscopy relies on the use of specific dyes or autofluorescent molecules, which may restrict its applicability to certain cell types (Bentahar *et al.*, 2024). Additionally, the equipment required for fluorescence microscopy can be expensive, and it requires very thin sectioning during sample preparation to avoid scattering of light if a confocal configuration is not used (Van Eeckhout *et al.*, 2021). Nevertheless, advancements in fluorescence microscopy, such as multispectral imaging (Bentahar *et al.*, 2024) and live-cell imaging techniques (Liu *et al.*, 2024), have enabled users to monitor dynamic physiological changes with greater precision. For example, Naim *et al.* (2021) have used X-ray fluorescence microscopy to visualize the nutrient acquisition and distribution dynamics in wheat leaves and a pathogen, allowing for a better understanding of the disease development in susceptible hosts.

While newer techniques such as light-sheet fluorescence microscopy and fluorescence lifetime imaging (FLIM) have shown great promise in plant stress studies, their use in wheat research is still in its infancy. These advanced methods have primarily been applied to model species or non-heat-stress conditions but hold potential for future wheat studies. For instance, light-sheet fluorescence microscopy was used to map the spatiotemporal distribution of superoxide dismutase FSD1 in Arabidopsis roots under salt stress, revealing subcellular localization patterns critical for understanding oxidative stress responses (Dvořák *et al.*, 2021). Similarly, FLIM has been used to assess virus-induced chlorosis in tobacco leaves, where shorter fluorescence lifetimes were linked to thylakoid membrane damage and chloroplast degradation (Lei *et al.*, 2017). Although not yet extensively applied to wheat, these techniques could be adapted to investigate how heat stress impacts wheat chloroplasts and cellular ROS management, offering early detection of stress-induced photosynthetic dysfunction and tissue damage.

In summary, many microscopy techniques, including LM, SEM, TEM, and fluorescence microscopy, have provided valuable insights into the anatomical and physiological responses of wheat leaves under heat stress. These methods remain essential for understanding how plants adapt to changing environmental conditions at the cellular and tissue levels. Integrated techniques built up on the fundamental technology could offer advantages such as higher resolution, higher dimension, and fewer artifacts. The integration with chemical composition analysis (e.g. FTIR and Raman spectroscopy) and mechanical property analysis (e.g. AFM) could further augment the information acquired by the imaging techniques. However, their limitations, particularly in terms of dynamic imaging and sample preparation, underscore the importance of adopting newer technologies to complement and enhance these microscopy methods.

## Recent technological advancements in studying leaf structure under heat stress

**Fig. 3.**
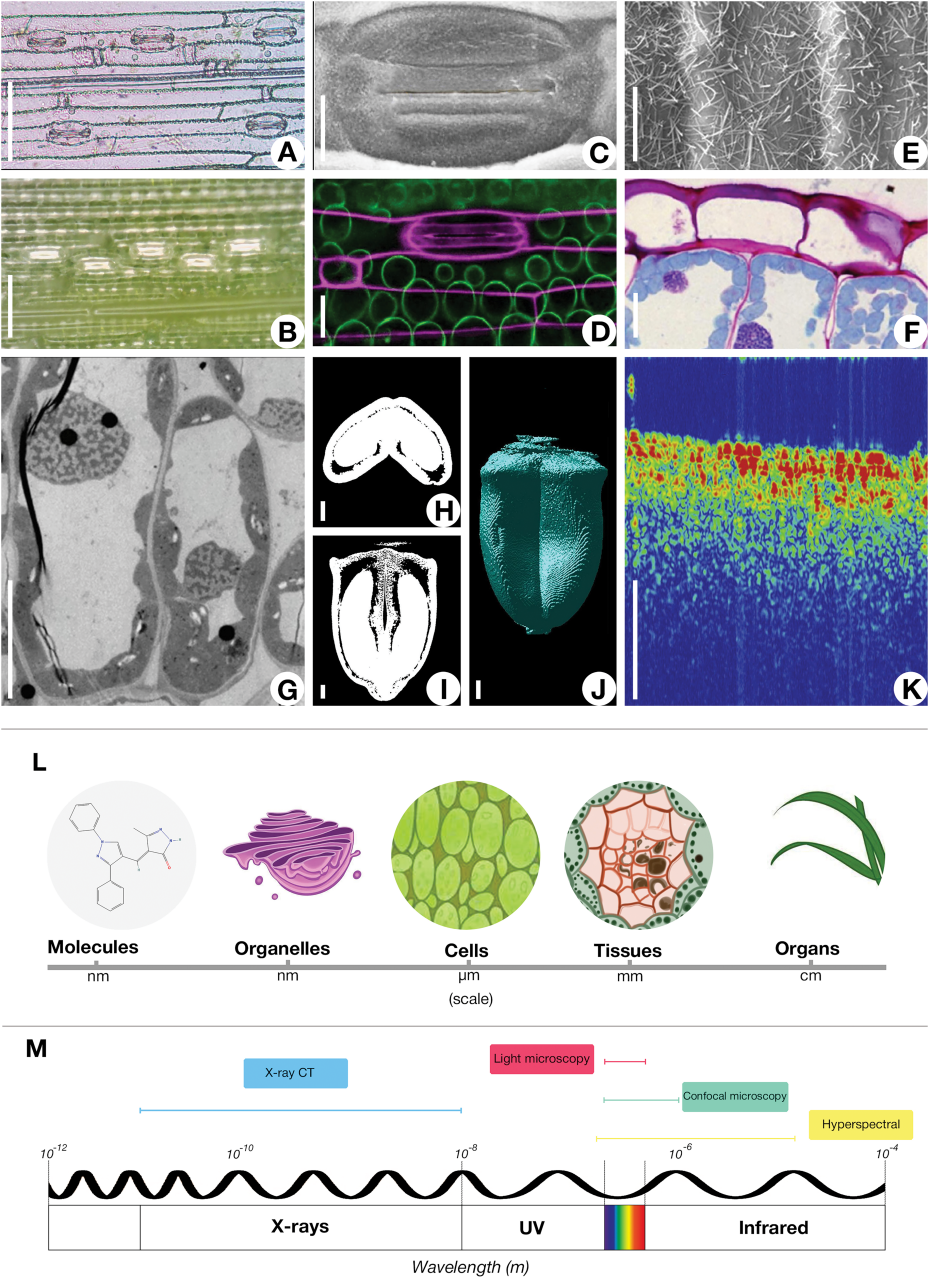
Images obtained using different types of microscopy techniques and their corresponding scales and wavelengths. (A) Wheat leaf epidermis imaged with light microscopy (source: authors). (B) Wheat leaf epidermis captured using a portable microscope (source: authors). (C) Wheat leaf epidermis visualized with scanning electron microscopy (Sehar *et al.*, 2021). (D) Wheat leaf stained with pseudo-Schiff propidium iodide and imaged using a confocal laser microscope (Lundgren *et al.*, 2019). (E) Trichomes on the wheat leaf observed with scanning electron microscopy (Jäger *et al.*, 2014). (F) Cross-section of a wheat leaf stained with periodic acid–Schiff and Coomassie Brilliant Blue, showing epidermis and mesophyll structures imaged with light microscopy (Jäger *et al.*, 2014). (G) Mesophyll cells of a wheat leaf visualized with transmission electron microscopy (Ouyang *et al.*, 2017). (H–J) Wheat seeds examined with X-ray imaging: transverse view (H), frontal view (I), and 3D view (J) (Le *et al.*, 2019). (K) Wheat leaf structure analysed with optical coherence tomography (Rateria *et al.*, 2019). (L) Scale of wheat leaf anatomical structures. (M) Spectrum of imaging techniques categorized by the light wavelengths used. Scale bars: 100 μm (A, B), 10 μm (C–G), 600 μm (H–K).

Recent advancements in imaging technologies have revolutionized the study of plant biology, overcoming many limitations of traditional microscopy. These innovative methods provide high-resolution, non-destructive imaging and enable real-time monitoring of cellular and tissue-level dynamics, leading to deeper insights into the structural and physiological mechanisms underlying plant resilience to heat stress. A comparative overview of wheat structures captured using various microscopy techniques, highlighting the varying size scales and electromagnetic wavelengths corresponding to these techniques, is provided in Fig. 3. This section explores the principles, applications, recent advancements, and limitations of three key technological approaches.

### Confocal laser scanning microscopy

CLSM has become a cornerstone of high-resolution imaging of biological samples, using a focused laser beam and pinhole optics to eliminate out-of-focus light and construct 3D images (Jonkman *et al.*, 2020; J. Zhu *et al.*, 2020). Unlike light microscopy, CLSM uses point-by-point laser scanning to generate detailed optical sections, which are reconstructed into 3D images (M’Saad and Bewersdorf, 2020). This technique is particularly effective for imaging fluorescently labelled structures, such as chloroplasts, cell walls, cuticular layers, and subcellular localization of proteins with sub-micrometre accuracy (Diel *et al.*, 2020; Khoshravesh *et al.*, 2022).

For wheat leaf anatomy, CLSM has been employed to investigate stomatal responses (Zubairova *et al.*, 2019), chloroplast morphology and mesophyll cell arrangement (D. Zhu *et al.*, 2021; Han *et al.*, 2023) under abiotic stresses. In one study, CLSM was used to examine mesophyll cell spatial arrangement in tobacco leaves transformed with wheat genes, shedding light on the effects of cellular organization on gas exchange efficiency under elevated temperatures (Ayadi *et al.*, 2011). However, the shallow imaging depth of CLSM and reliance on fluorescent markers on non-fluorescent molecules limit its application to thin tissues and necessitate complex sample preparation (M’Saad and Bewersdorf, 2020; Yuan *et al.*, 2020).

To overcome these limitations, advanced techniques such as Raman confocal microscopy and hyperspectral confocal fluorescence microscopy have been developed. Raman confocal microscopy combines Raman spectroscopy with CLSM, capturing a full Raman spectrum at each image pixel and achieving lateral resolution down to the diffraction limit (half the excitation wavelength) (Gomes da Costa *et al.*, 2019). This non-invasive technique captures real-time biochemical changes, such as carotenoid degradation and phenolic compound accumulation, which are critical markers of stress response. In maize, this system has been employed to monitor carotenoid degradation as a drought stress marker (Altangerel *et al.*, 2021). Applied to wheat leaves, it could reveal metabolic changes under heat stress, such as phenolic compound accumulation and ROS detoxification pathways. Similarly, hyperspectral confocal fluorescence microscopy extends the capabilities of CLSM by providing precise spectral detection with minimal interference. Conventional fluorescence hyperspectral imagers suffer from low resolution and blurry images due to axial interference, which can be eliminated with CLSM for higher signal-to-noise ratio and precise spectral detection of a single fluorescence point (Luo *et al.*, 2021). This method has been utilized to analyse chlorophyll stability and lipid content in live, unstained cells (Hanson *et al.*, 2014; Luo *et al.*, 2021). By visualizing chlorophyll fluorescence lifetimes and lipid distribution, this technique could facilitate a deeper understanding of photosynthetic dysfunction and membrane integrity in wheat leaves under heat stress. Overall, CLSM and its associated techniques can provide a powerful toolkit for non-invasively studying both structural and biochemical responses of wheat leaves to heat stress, advancing our understanding of plant resilience at the cellular level.

### Tomographic imaging techniques

Tomographic imaging techniques, including CT and OCT, have advanced the non-invasive study of plant structures. Both methods reconstruct 3D images from 2D cross-sectional data, enabling the visualization of complex anatomical features without damaging the tissue. CT generates high-resolution images of internal structures based on variations in X-ray attenuation (Piovesan *et al.*, 2021). It has been widely applied to study vascular networks and tissue architecture (Mathers *et al.*, 2018; Du *et al.*, 2022). In wheat, CT has been used to develop high-throughput, non-destructive methods for assessing yield-related traits under heat and drought stress (Schmidt *et al.*, 2020; Zhou *et al.*, 2021). This includes precise measurement of spike architecture, seed morphology, and stress-induced seed deformities, such as shrivelling (Le *et al.*, 2019; Schmidt *et al.*, 2020). Additionally, CT has been used to assess xylem vulnerability to embolism under abiotic stresses, revealing water use efficiency and vascular integrity (Brodersen *et al.*, 2010; Cochard *et al.*, 2015). This technique is also able to uncover wheat root architecture, providing insights into rhizosphere spatial relationships and gene expression patterns (Khalil *et al.*, 2020). Despite these capabilities, the resolution of CT is generally lower than that of advanced microscopy techniques like CLSM, which limits its ability to resolve fine cellular ultrastructures (Rawson *et al.*, 2020; Piovesan *et al.*, 2021). Moreover, prolonged X-ray exposure can cause cellular damage in live tissues, underscoring the need for optimized imaging protocols (Petruzzellis *et al.*, 2018; Withers *et al.*, 2021). Advances such as X-ray micro-computed tomography (μCT) have significantly improved resolution to sub-micrometre levels, making it suitable for visualizing reproductive development in wheat spikes and correlating heat-induced anatomical changes with phenological traits (Dhondt *et al.*, 2010; Piovesan *et al.*, 2021).

On the other hand, OCT uses low-coherence light to capture images based on the scattering properties of biological tissues (Anna *et al.*, 2019). It is particularly suited for studying surface-level structures, such as stomatal dynamics and cuticle thickness, with high temporal resolution (de Wit *et al.*, 2020). OCT has been used to quantify cuticle thickness and monitor changes in leaf water content, both of which are critical for plant stress tolerance (Licaj *et al.*, 2024). In tobacco leaves, OCT revealed early chloroplast alterations during the harpin-induced hypersensitive response, detecting changes in thylakoid membrane structure before visible cell death occurred (Boccara *et al.*, 2007). For wheat, OCT could facilitate the early detection of stress-related disruptions in chloroplast function and photosynthesis, aiding in the understanding of heat tolerance mechanisms. Although the shallow penetration depth of OCT limits its application to surface structures, advancements such as biospeckle OCT (bOCT) enhance its utility. For instance, bOCT was used to assess cytoplasmic streaming and internal leaf dynamics in response to gibberellic acid application in Chinese chives, revealing physiological changes undetectable by structural OCT imaging (Rajagopalan *et al.*, 2020). This capability could be adapted for assessing rapid, non-destructive physiological responses such as cytoplasmic streaming and biochemical activity to heat stress in wheat.

Besides CT and OCT, other tomographic techniques, such as positron emission tomography (PET) and laser ablation tomography (LAT), have emerged as powerful tools for analysing plant stress responses, offering unique insights into physiological and structural changes. PET is a non-invasive imaging method that uses radiolabelled tracers to study dynamic processes such as carbon and water transport within leaves (Antonecchia *et al.*, 2022). It is particularly effective for monitoring photosynthate distribution and understanding how heat stress impacts nutrient flow and metabolic pathways (Galieni *et al.*, 2021). For example, PET could identify heat-induced disruptions in source–sink relationships, providing insights into the carbon allocation dynamics of wheat under stress (Karve *et al.*, 2015). LAT, on the other hand, employs high-powered laser pulses to sequentially ablate and image plant tissues, creating high-resolution 3D reconstructions of internal structures (Strock *et al.*, 2022). It has been used to visualize xylem networks and assess changes in vascular architecture under heat stress (Strock *et al.*, 2019, 2022). LAT offers valuable insights into how heat affects water transport efficiency and xylem integrity in wheat, revealing vulnerabilities in the plant hydraulic system. The integration of tomographic techniques offers new possibilities for holistic analysis. For example, magnetic resonance imaging–PET systems enable simultaneous imaging of water transport and carbon allocation dynamics, capturing the translocation patterns of photoassimilates under stress (Jahnke *et al.*, 2009). Taken together, these integrated methods provide a holistic perspective, merging anatomical and physiological (e.g. water use efficiency, carbon partitioning) insights that could significantly advance our understanding of wheat responses to heat stress.

### Live tracking and monitoring of stomatal dynamics

Recent advancements in microscopy have enabled real-time monitoring of stomatal movements, providing a deeper understanding of how wheat leaves modulate water loss and gas exchange under heat stress. These tools reveal dynamic responses to fluctuating environmental conditions and offer valuable insights for breeding heat-tolerant wheat varieties.

High-speed video microscopy captures real-time stomatal movements with high temporal precision (Sun *et al.*, 2021). This method is particularly effective for studying rapid stomatal closure and opening events, which are critical for regulating water use efficiency and photosynthesis under heat stress (Sai *et al.*, 2023). For instance, Pathoumthong *et al.* (2023) demonstrated the use of handheld video microscopy integrated with ML algorithms for rapid, non-destructive phenotyping of stomatal traits like number, size, and aperture. This method facilitates large-scale data acquisition, directly supporting wheat breeding efforts for heat resilience. Additionally, ML algorithms facilitate automated detection and quantification of stomatal apertures from video data, as demonstrated by Liang *et al.* (2022) and Sun *et al.* (2023). Furthermore, Toda *et al.* (2021) developed an ML-driven image-analysis platform using a USB camera and a Jetson Nano GPU-supported device to detect traits such as stomatal density and size directly from wheat leaf imprints. This rapid screening tool allows high-throughput assessment of gas exchange traits, providing valuable insights for wheat heat tolerance studies.

By capturing the longwave radiation emitted by an object and directing it onto a temperature-sensitive detector, thermal imaging enables the observation of leaf surface temperature. This allows for the non-invasive and contactless monitoring of stomatal conductance and dynamics—high leaf temperatures are closely linked to stomatal closure (Sirault *et al.*, 2009; Elsayed *et al.*, 2017). Additionally, thermal imaging is useful in assessing water stress and water usage, as water deficit leads to stomatal closure, resulting in an increase in temperature (Sirault *et al.*, 2009; Elsayed *et al.*, 2017). In wheat, thermal imaging can be applied to screen for varieties that are tolerant to abiotic stresses such as heat, drought, and salinity (Sirault *et al.*, 2009; Pineda *et al.*, 2021). Furthermore, thermography can now be integrated with ML, RGB, and hyperspectral technologies to expand its agricultural applications (Gutiérrez *et al.*, 2018; Osroosh *et al.*, 2018; Krishna *et al.*, 2021).

Microfluidic platforms, when integrated with AI/ML tools, provide precise environmental control (e.g. humidity, light, temperature) for monitoring population-level stomatal dynamics in real-time (Yanagisawa *et al.*, 2021). For instance, microfluidic systems have demonstrated how groups of stomata synchronize their responses to fluctuating humidity and heat stress (Koman *et al.*, 2017). However, these set-ups are limited by small sample sizes and challenges in simulating field-like conditions. Efforts to integrate microfluidics with high-resolution confocal microscopy have shown promise but remain challenging to scale for field applications (Yanagisawa *et al.*, 2021).

Overall, live tracking and monitoring techniques have significantly advanced our understanding of how stomatal dynamics and population-level responses in wheat are regulated under heat stress, offering critical insights into the mechanisms governing plant water use and gas exchange. While high-resolution and live imaging techniques can provide a wealth of anatomical and physiological information about wheat leaf responses to heat stress, the sheer volume and complexity of the data generated present new challenges.

## Combined imaging and predicting technologies with artificial intelligence and machine learning

**Fig. 4.**
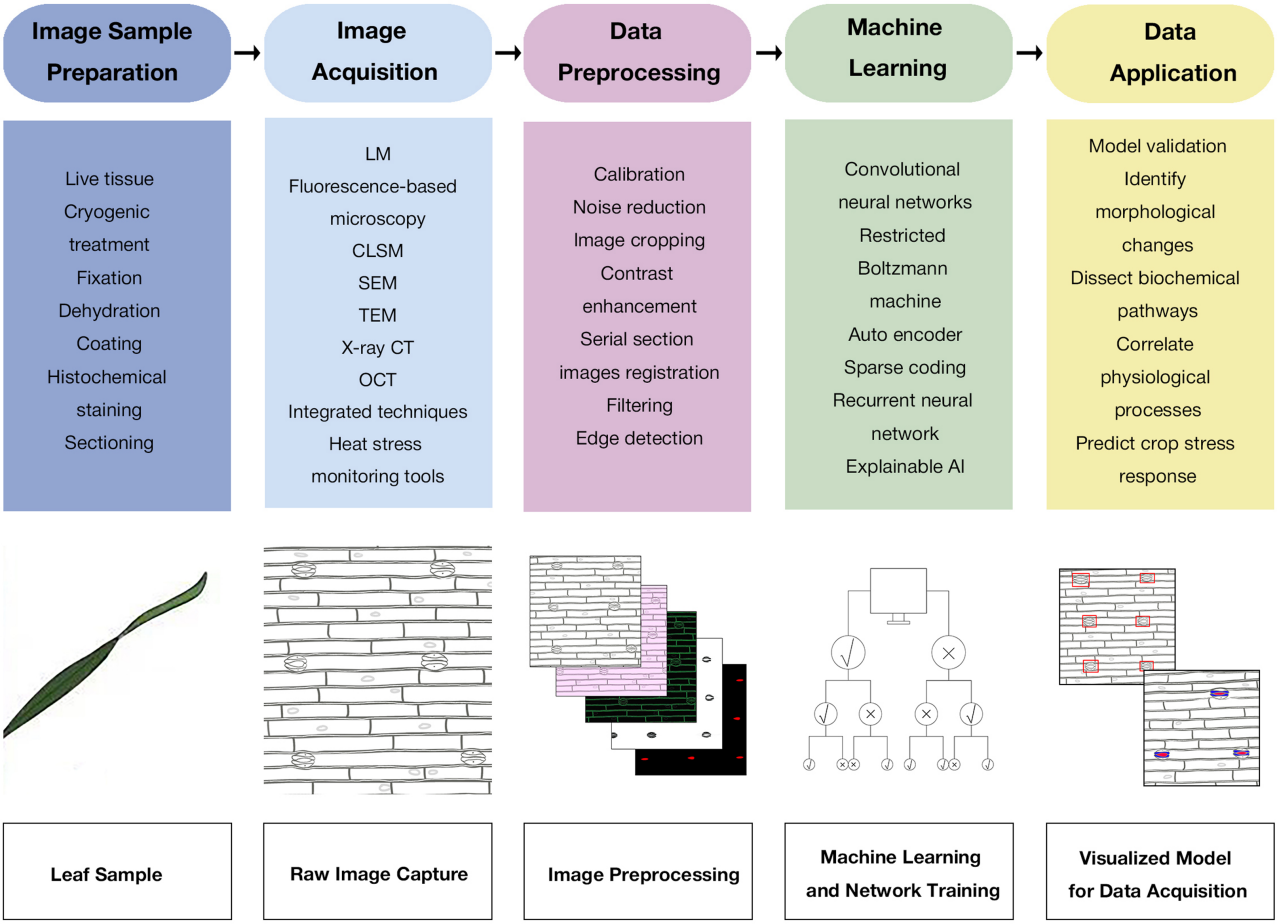
Workflow of integration of imaging technologies with artificial intelligence/machine learning for plant phenotyping. CLSM, confocal laser scanning microscopy; CT, computed tomography; OCT, optical coherence tomography; SEM, scanning electron microscopy; TEM, transition electron microscopy.

The integration of advanced imaging technologies with AI/ML is transforming plant science and crop breeding by enabling accurate, high-throughput phenotyping and predictive modelling of plant performance. These methodologies bridge the gap between phenotypic data and environmental stress responses, offering new opportunities for studying wheat heat tolerance. An integrated workflow for combining imaging technologies with AI/ML in plant phenotyping is illustrated in Fig. 4. This structured methodology improves data acquisition, image interpretation, and phenotypic trait quantification, making it scalable for large datasets from field trials and controlled environments. This section explores AI/ML algorithms in image analysis and their integration with imaging technologies to predict plant performance.

### Artificial intelligence and machine learning algorithms for image analysis

Recent advances in AI and ML, particularly deep learning (DL) and computer vision (CV), have revolutionized image analysis in plant science. DL is a subset of ML that mimics the neural networks of the human brain to process large volumes of complex data (C. Zhu *et al.*, 2021). It is particularly effective in identifying patterns and relationships in unstructured datasets, such as images, and improving the accuracy over time (Bian *et al.*, 2022). CV, a branch of AI, leverages the DL algorithms and enables machines to interpret and analyse visual data. Together, these tools have transformed image-based phenotyping by automating the analysis of high-resolution data and extracting meaningful insights into plant structure and function (Toda *et al.*, 2021; Liang *et al.*, 2022). CV tasks typically involve image recognition, visual tracking, semantic segmentation, and image restoration (Chai *et al.*, 2021). Convolutional neural networks (CNNs), a key architecture in CV, excel at tasks like stomatal detection and pore measurement (Zhang *et al.*, 2024). For instance, StomaAI uses DL to measure stomatal pores and densities in barley and Arabidopsis, enhancing throughput and achieving human-level accuracy (Sai *et al.*, 2023). Similarly, StomataTracker analyses video data through DL to monitor stomatal behaviour in wheat, revealing nocturnal patterns that affect water conservation (Sun *et al.*, 2023).

Machine learning models such as the Cascade object detection (COD) algorithm have also been tailored to quantify stomatal dynamic traits and eccentricity in response to fluctuating environmental conditions (Jayakody *et al.*, 2017). The integration of the ML and DL algorithms with non-destructive imaging techniques, such as CLSM (Möller *et al.*, 2017), μCT (Rippner *et al.*, 2022), and SEM (Bhugra *et al.*, 2019), provides new avenues to understand heat-induced changes in features such as mesophyll structure and water transport mechanisms. The AI-based image analysis ensures that these observations are robust, reproducible, and scalable, making it feasible to analyse large datasets from field trials and controlled experiments for wheat heat tolerance study.

### Development of predictive models for crop performance

The integration of AI and ML with high-resolution imaging technologies has opened new avenues for predictive modelling in crop science. These models simulate complex physiological and anatomical traits across multiple scales, from cellular processes to whole-plant systems, providing valuable insights into crop performance under stress conditions, including heat (Xiao *et al.*, 2023; Zhang *et al.*, 2024).

Many predictive models are grounded in differential equations, which describe dynamic processes such as water transport, photosynthesis, and stomatal regulation. The models that are built on differential equations are pure mathematical models, using parameterized inputs from physiological measurements. For instance, Richard’s equation models water flow through soil and plant tissues, while the Doussan model simulates water movement within xylem vessels (Doussan *et al.*, 2006). Both have been implemented in platforms like CRootBox to visualize xylem vulnerability and predict water-use efficiency at the tissue and organ levels (Schnepf *et al.*, 2018). These models estimate key parameters such as hydraulic conductance, water potential, and root water uptake rates (Koch *et al.*, 2018). At the tissue scale, the Tardieu–Davies model integrates environmental variables with stomatal conductance to simulate transpiration and hydraulic conductance. This model can be adapted to wheat to assess how stomatal regulation influences gas exchange under heat stress, providing genotype-specific insights for breeding programmes (Tardieu *et al.*, 2015).

Extending to cellular processes, models like eLeaf integrate imaging data with biochemical and biophysical inputs to simulate photosynthesis. By simulating light and CO_2_ distribution within leaf tissues, these models quantify traits such as light absorption and CO_2_ diffusion within leaf tissues, offering insights into how anatomical adaptations influence carbon assimilation and photosynthetic resilience (Xiao *et al.*, 2023). Unlike purely mathematical models, eLeaf uses real imaging inputs, such as mesophyll and chloroplast arrangement, to parameterize its simulations. 3D microscale models take this further by incorporating light propagation and internal CO_2_ transport within leaf tissues to predict photosynthetic rates. Such models have been successfully applied to tomato plants, demonstrating how variations in leaf anatomy impact photosynthetic efficiency under fluctuating environmental conditions (Ho *et al.*, 2016). Applied to wheat, these models could help optimize leaf structure to enhance photosynthesis and yield under heat stress.

### Challenges in integrating artificial intelligence and machine learning models for image processing and predictive models

Despite promising advancements, several challenges limit the full potential of AI/ML applications in microscopic image analysis and predictive models. One primary obstacle is the quality and heterogeneity of imaging data. Variability in image resolution, lighting conditions, and tissue transparency can negatively impact ML algorithm performance, requiring extensive data preprocessing and normalization (Khoshravesh *et al.*, 2022). Furthermore, generalizing models across diverse wheat varieties and environmental conditions demands large, annotated datasets, which are often difficult to compile (Zhang *et al.*, 2024). Another significant challenge lies in the interpretability of AI models. While DL excels at pattern recognition, it often operates as a ‘black box’, limiting the biological significance of its outputs (Sai *et al.*, 2023). Explainable AI (XAI) frameworks are being developed to address this issue. For example, SHapley Additive exPlanations (SHAP) values measure the contribution of individual variables to predictions, offering insights into how specific traits influence outcomes. Singh *et al.* (2022) utilized SHAP to identify critical spectral wavelengths for nitrogen estimation in wheat, linking model predictions to physiologically meaningful features.

The integration of AI/ML with physiological data also requires interdisciplinary collaboration among plant biologists, data scientists, and environmental modellers (Zhang *et al.*, 2024). Limited access to computational resources presents another hurdle. Training complex models demands high-performance computing infrastructure, and while cloud-based solutions are emerging, accessibility remains an issue for many institutions (Strock *et al.*, 2019; Khoshravesh *et al.*, 2022). Efforts to democratize AI/ML tools through user-friendly, open-source platforms are ongoing, aiming to make these powerful methods more accessible. Moving forward, optimizing AI/ML for practical applications in crop improvement will involve overcoming these challenges and ensuring their usability in diverse research settings.

## Conclusions and future perspectives

Significant advancements in high-resolution imaging technologies and the integration of AI/ML have greatly expanded our ability to study wheat leaf anatomy and physiological responses to heat stress. However, challenges remain. Advanced imaging techniques, such as CLSM, CT, and OCT, face trade-offs between resolution and tissue penetration depth, while the high cost and technical expertise required for these technologies limit their accessibility (Jonkman *et al.*, 2020; Zhang *et al.*, 2024). Additionally, processing and storage of large datasets generated by these imaging systems require robust computational infrastructure, which is often unavailable in many academic settings (Bian *et al.*, 2022; H. Zhang *et al.*, 2023).

Throughput limitations further hinder scaling these technologies to large-scale phenotyping, while multimodal imaging, such as combining hyperspectral data with CLSM, presents integration challenges due to differences in spatial resolution, imaging depth, and data formats (Jahnke *et al.*, 2009; Altangerel *et al.*, 2021). Variability in sample preparation, imaging settings, and data processing can lead to inconsistencies, making it difficult to compare findings across studies or replicate experiments (Harwood *et al.*, 2020; Guo *et al.*, 2022). Standardized protocols and automated workflows for data harmonization and analyses remain a priority for advancing these methods. Moreover, translating findings from high-resolution imaging studies into practical agricultural applications remains complex due to environmental variability and cost considerations. Field-deployable sensors and remote sensing technologies can complement controlled environment studies, enabling real-time monitoring of plant responses to heat stress at a population level (Liang *et al.*, 2022). Collaboration among plant breeders, agronomists, and data scientists is critical to develop scalable, cost-effective tools for identifying heat-tolerant genotypes.

The future of imaging-based research in plant science lies in a more holistic and integrated approach. The convergence of high-resolution imaging technologies with physiological and multi-omics studies holds immense promise (Wang *et al.*, 2020). For example, combining imaging data with genomics, transcriptomics, proteomics, and metabolomics could provide a comprehensive view of how anatomical changes under heat stress are linked to underlying genetic traits and molecular pathways (D. Zhu *et al.*, 2021; Guo *et al.*, 2022). Such integrative studies could identify novel biomarkers for heat tolerance and uncover the genetic basis of adaptive traits, informing targeted breeding strategies. The integration of AI/ML into imaging workflows has revolutionized plant phenotyping, but there is still room for improvement. XAI frameworks are emerging as a promising solution, offering a way to interpret how specific anatomical traits influence wheat heat stress tolerance. Future research should focus on refining these AI models to ensure they are robust and generalizable across diverse environmental conditions and plant genetic backgrounds. This involves developing algorithms capable of handling heterogeneous and noisy datasets, a common challenge in field-based phenotyping studies. Additionally, integrating ML models with mechanistic and biophysical simulations, such as those predicting water transport and carbon allocation, could create more comprehensive frameworks for understanding heat stress responses. It is highly likely that the future approach will have a combination of high-throughput phenotyping, multimodal data integration, and advanced computational modelling. As imaging technologies continue to evolve, so too will our ability to link anatomical and physiological traits with genetic and environmental factors, paving the way for the development of climate-resilient wheat crops. Continued investment in interdisciplinary initiatives and user-friendly technologies will ensure the next generation of researchers are equipped to harness these tools effectively.

## Data Availability

Supporting image data analysed as part of this review were obtained from previously published studies, which are cited appropriately within the text at relevant places.
